# What do Australian health consumers believe about commercial advertisements and testimonials? a survey on health service advertising

**DOI:** 10.1186/s12889-020-10078-9

**Published:** 2021-01-07

**Authors:** ACL Holden, S. Nanayakkara, J. Skinner, H. Spallek, W. Sohn

**Affiliations:** 1 The University of Sydney School of Dentistry, Faculty of Medicine and Health, 2-18 Chalmers Street, Sydney, NSW 2010 Australia; 2 The University of Sydney School of Public Health, Faculty of Medicine and Health, University of Sydney, Edward Ford Building (A27), Sydney, NSW 2006 Australia; 3 The Poche Centre for Indigenous Health, Faculty of Medicine and Health, University of Sydney, Edward Ford Building (A27), Sydney, NSW 2006 Australia

**Keywords:** Advertising, Commercialism, Decision-making, Professional regulation, Health policy

## Abstract

**Background:**

There has been little examination of consumer attitudes towards the commercial advertising of healthcare services in Australia and how marketing is used by consumers in their health decision-making. In this research, we examined how consumers reported commercial advertising helped them to understand the health services available to them and the influence that marketing had upon their choices.

**Methods:**

A survey instrument using a Likert scale to indicate agreement or disagreement with 21 questions was developed using qualitative interviews before being distributed online within Australia. Sampling of participants was stratified by age, gender and location. The results were subjected to statistical analysis with Spearman Rank Correlation test being used for bivariate analysis.

**Results:**

One thousand five hundred sixty-four complete surveys were collected. The results revealed certain consumer beliefs, for example; the title of ‘Dr’ was believed to indicate skill and high levels of training (81.0%), with 80.3% agreeing incorrectly that use of the title was strictly regulated. Participants reported to have a higher confidence in their own abilities (71.2%) than the public (52.8%) in assessing health advertising. The level of self-confidence increased with higher education level and decreased by age (*p* < 0.05). Testimonials were reported to be lacking in reliability (67.7%) and that they should not be used in healthcare in the same manner as they are used in other industries. Only 44.8% of participants reported that they felt confident to spot a review that was not written by a genuine user of a service.

**Conclusions:**

The data demonstrated that many health consumers felt that while commercial health advertising was helpful, it was also confusing, with many participants also holding mistaken beliefs around other elements of commercial health advertising. While the advertising of healthcare services might have educational effects and be superficially empowering, advertising is primarily intended to sell, not educate. This research demonstrates that there is significant potential for healthcare advertising to mislead. Future developments in regulatory health advertising policy, and the related ethical frameworks developed by professional healthcare associations, need to consider how the consumers of healthcare services might be better protected from misleading and predatory advertising practices.

**Supplementary Information:**

The online version contains supplementary material available at 10.1186/s12889-020-10078-9.

## Background

### Professional attitudes

The earliest modern “Code of Ethics” from the American Medical Association [1] clearly sets out how advertising was situated within the obligations of the medical professional of the period;

It is derogatory to the dignity of the profession to resort to public advertisements, or private cards, or handbills, inviting the attention of individuals affected with particular diseases – publicly offering advice and medicine to the poor gratis, or promising radical cures; or to publish cases and operations in the daily prints, or suffer such publications to be made; to invite laymen to be present at operations, to boast of cures and remedies, to adduce certificates of skill and success, or to perform any similar acts. These are the practices of empirics, and are highly reprehensible in a regular physician [1]. (page 11)

At this early stage in the development of the modern medical profession’s identity and professional jurisdiction, advertising was perceived to be antithetical to professional conduct and viewed as encouraging competition within medicine and the provision of care to the public.

The organised and regulated health professions largely maintained this position for much of the 19th and 20th Centuries, with professional associations being critical of practitioners who engaged in the advertisement of their professional services. In the USA, professional prohibitions were challenged and defeated in a case involving the legal profession, which impacted all organised professions where advertising prohibitions were in force [2]. Whilst the legal prohibitions were removed to advertising, professional associations have remained cautious of healthcare advertising, being sceptical of its value [2].

In twenty-first Century health practice there is an increasing societal expectation that practitioners will have an online presence [3]. The rapid expansion of social networking using online platforms, such as Facebook and Instagram, has made information about health services far more accessible. Increasing demand from health consumers to be able to find information about health services has led to practitioners being increasingly visible, with third-party agencies making it possible to rate and comment on consumer experience and engagement.

In Australia, prior analysis of advertising policy and positions demonstrated that professional groups are still cautious about the impacts and utility of advertising in the health arena [4]. The Australian Medical Association’s position statement; Advertising and Public Endorsement 2004 [5] discusses concerns that commercial health advertising may not present relevant information that aids informed decision making. While commercially-oriented health advertising is permitted in Australia, it is regulated by a legal framework which seeks to protect the public.

### Legislative framework and restrictions in Australia

With each state and territory across Australia enacting similar legislation, the Health Practitioner Regulation National Law brings alignment to how health practitioners regulated by the 15 National Boards are administered and licensed. An area where uniformity exists is the prohibition of certain advertising strategies and methods that relate to regulated health services. The New South Wales legislation - Health Practitioner Regulation National Law 86a 2009 (NSW) – states in section 133:

A person must not advertise a regulated health service, or a business that provides a regulated health service, in a way that: (a) is false, misleading or deceptive or is likely to be misleading or deceptive; or (b) offers a gift, discount or other inducement to attract a person to use the service or the business, unless the advertisement also states the terms and conditions of the offer; or (c) uses testimonials or purported testimonials about the service or business; or (d) creates an unreasonable expectation of beneficial treatment; or (e) directly or indirectly encourages the indiscriminate or unnecessary use of regulated health services.

Of these prohibitions, the restriction of the use of testimonials has been recognised to be particularly divisive. A range of views relating to the use of testimonials in advertising can be found within the public submissions to the review of the National Registration and Accreditation Scheme published by the Australian Health Ministers Advisory Council [6]. Testimonials persist as a mode of advertising health services that may be abused. Research conducted in 2017 found that within the study sample, the majority of dental practices advertised used testimonials in a way that contravened the National Law [7]. Online testimonials do not contravene the National Law when they are provided by a third party over which the practitioner or health service being reviewed has no control. Recent research exploring the use of patient narratives in healthcare more broadly found that the use of narratives in healthcare were potentially supportive to other patients, enhancing of patient empowerment and had the power to educate and inform both patients and healthcare practitioners [8]. Despite the promise that patient narratives might have within a therapeutic context, there is little exploration of how they might be used within a commercial context, to either mislead, educate or empower patients and consumers about healthcare services. Research examining patient acceptance of misleading claims made by pharmaceutical companies would suggest that consumers are highly susceptible to deceptive claims and tactics [9]. This research will examine how testimonials and other commercial advertising of healthcare services may impact the way that healthcare consumers make decisions about which services to access.

Whilst the National Law applies to the practice of health professionals who are registered by the Australian Health Practitioner Regulation Agency (AHPRA), other, non-registered health practitioners have voluntarily adopted some or all of the National Law’s advertising restrictions. The Dietitians Association of Australia (DAA) does not permit its members to use testimonials, stating; “As a self-regulated profession, DAA aims to maintain or exceed the standards and requirements for registered professions” [10]. The NSW Code of Conduct for unregistered health practitioners states, “A health practitioner must not make claims, either directly or in advertising or promotional material, about the efficacy of treatment or services provided if those claims cannot be substantiated.” [11]. Similar codes of conduct exist in South Australia and Victoria, but at the time of writing, other states and territories have not yet produced regulatory frameworks for health practitioners who fall outside the National Law.

Other work examining the future of heath advertising in Australia discusses how other agencies contribute to the framework of regulation surrounding the advertisement of certain types of health services [4]. The Australian Competition and Consumer Commission (ACCC) and the Therapeutic Goods Administration (TGA) both contribute to the overall framework along with the National Law.

### The healthcare patient as a consumer

Pellegrino [12] suggested that those who have not been forced to examine the frailty of good health when consuming healthcare would be more likely to have a market-based attitude to selecting which healthcare services they might access. In this paradigm, patients are in many ways no different to the consumers of any other type of service; free to shop around, compare price and make decisions about which services to access based on personal preference. Pellegrino was discussing the medical profession when this inference was drawn; it is likely that for other health services such as dentistry, chiropractic and traditional Chinese medicine, the commercialised nature and lack of public funding towards these services is likely to further accentuate this market-based ethic. This contrasts with instances where the acutely sick and unwell access services. They are faced with the realities of their own mortality and are likely to be far more restricted in what choices are available to them and whether to have treatment. The primary focus of this survey was to understand how respondents interacted with, and assessed, the claims made within the advertising of health services, and how they evaluated the statements made by purported consumers within testimonials in relation to their experiences of specific health services. This research is interested in advertising used as a commercial tool in order to attract engagement with services, rather than any social marketing or health promotion strategy. Understanding how health services which are reliant on attracting consumers within a free-market might impact consumer attitudes and decision-making through their advertising is important for the development of future health policy.

Baudrillard [13] noted that the finest consumer object was the body, with bodies having value as status and prestige symbols. It is therefore difficult to define a distinction between healthcare services which provide relief from pain, disease and suffering, and services which are provided by healthcare providers, but that are oriented towards making oneself better than well. Commercialised healthcare may frustrate this process through advertising which makes individuals feel obliged to undergo services through highlighting societally defined flaws in appearance. The way that this has occurred in the dental industry has been described by Otto [14] and Holden [15], where the public are provided with oral healthcare services that they did not perceive they needed before it was ‘sold’ to them. It is difficult to draw a definitive line between treatments which are solely elective; provided to enhance appearance and cosmetic outcomes, and those which are primarily concerned with advancing health and which have a secondary purpose of improving aesthetics.

With this research, we develop a greater understanding of how Australian healthcare consumers respond to advertising related to regulated health services and how members of the public evaluate and use health advertising to make decisions with special emphasis on testimonials as they are perceived as being contentious within the healthcare arena. Prior research examining how consumers assess restaurant reviews suggested that consumers find extreme ratings left online to be more useful than moderate reviews [16]. It is unclear whether these findings are translatable to healthcare services. Healthcare claims may be made in a way that is misleading or confusing to healthcare consumers. We present insights on how consumers perceive their own abilities to evaluate claims in advertising, as well as how consumers perceive and evaluate the expertise of health professionals.

## Methods

This study was given ethical approval by the human research ethics committee of the University of Sydney following full review (project number: 2019/168).

This research used qualitative interviews with health consumers to inform the development of an online survey which was then distributed using ﻿an electronic survey program (Qualtrics, Inc., Provo Utah, USA). Participants engaged in the qualitative development of the survey were recruited through adverts placed in four dental surgeries across Central and Western Sydney. The information shared by the nine participating health consumers allowed the research team to develop recurring themes relating to healthcare service advertisements and testimonials. These themes relate to how consumers use advertising to assess and evaluate which services they choose to access. The interviews also explored the attitudes and beliefs of health consumers towards health advertising.

A survey consisting of 21 items was developed based on the information from the qualitative interviews. The survey was presented with participants being asked to rate their agreement with the survey statements from strongly disagree to strongly agree in a 5-point Likert scale. Following initial development of the survey questions by the research team, a panel of expert academics in health ethics and law independent from the research team provided feedback on the questions. Established methods were used to finetune the survey instrument [17] and pilot test it using a retrospective think-aloud protocol [18] which established how participants were likely to interpret the questions being asked of them when responding to the survey. Pilot testing was completed after consensus developed following three pilot-testers providing feedback on how they interpreted the survey statements. Those individuals providing feedback were all from non-healthcare backgrounds. The final refined survey questions are presented as an additional file in the supplementary information. The survey also included questions about participants’ demographic information: location in Australia, age, gender, education level, language spoken at home, ethnicity and Aboriginal and Torres Strait Islander status.

Sampling for the purposes of sending out a survey were carried out through the utilisation of a sampling and distribution company, Qualtrics, allowing the survey to be sent out through their online distribution platform to a broad range of the population. The sample of participants recruited was stratified using age and gender quotas taken from census data and GeoIP in Australia.

Participants were recruited through actively managed, double-opt-in market research panels and social media. Qualtrics uses panel aggregation, meaning that a wide range of participants were sourced to take part in this survey. Participants were required to opt-in to take part twice, with those who only did so once not being invited to participate in the research. Participants sampled by Qualtrics were incentivised to take part in this research at a level commensurate with the time taken to complete the survey.

Statistical analysis of quantitative data from the participants was performed using IBM SPSS Statistics (version 23, IBM SPSS Inc., Chicago, IL). Data frequencies were summarized using percentages and cumulative percentages. Spearman Rank Correlation test was used for bivariate analysis. The reliability of the Likert scale survey instrument was assessed based on the internal consistency analysis (Cronbach’s alpha). For all the analyses, *p* ≤ 0.05 was considered as statistically significant.

## Results

### Demographics

The survey received 1564 responses, with a response rate of 49%. The response rate represents the proportion of those sent the invitation to participate in the survey who accepted, with 69% of those accessing the survey completing the questionnaire and passing screening criteria and eligibility checks. For example, participant responses that were provided in less than half the median response time were excluded from the sample. Approximately one third of the participants were from New South Wales while the Northern Territory had the lowest number of participants. Overall there was representation from all the 6 states and 2 territories. Over 90% of the participants used English as their main language at home. The demographic characteristics of the participants are summarised in Table 1. The distribution of males and females and the age groups in this cohort was similar to that of Australian society based on 2016 census data [19]. However, there was slightly lower representation from age groups over 75 years. Participants with an Indigenous background (Aboriginal, Torres Strait Islander or both) comprised 3.7% of the total responses. A quarter of the participants had a degree at Bachelor-level and over 85% had an education level at least year 12 or equivalent. Over 78% of the participants were white in ethnicity with South East Asians being the second most common ethnicity (7.2%).

**Table 1:** Demographic characteristics of participants

	Frequency (*n* = 1564)	Percentage
Gender		
Male	764	48.8
Female	799	51.1
Other	1	0.1
Age		
18–24	242	15.5
25–34	276	17.6
35–44	259	16.6
45–54	255	16.3
55–64	228	14.6
65–74	255	16.3
75–84	45	2.9
85+	4	0.3
Indigenous status		
Aboriginal	40	2.6
Torres Strait Islander	15	1.0
Neither	1507	96.4
Both	2	0.1
Ethnicity		
White	1221	78.1
Asian (South-East)	112	7.2
Indian	57	3.6
Asian (North-East or Chinese)	60	3.8
Pakistani	10	0.6
Arabic	12	0.8
African	10	0.6
Other	55	3.5
Mixed	27	1.7
Education		
Less than Year 12 or equivalent	232	14.8
Year 12 or equivalent (HSC/Leaving certificate)	281	18.0
Vocational Qualification	266	17.0
Associate Diploma	109	7.0
Undergraduate Diploma	74	4.7
Bachelor degree (including honours)	393	25.1
Masters degree	111	7.1
Postgraduate Diploma (includes graduate certificate)	76	4.9
Doctorate	22	1.4

### 21-item questionnaire: responses, reliability and correlation

Table 2 summarizes the percentage responses to the 21 questions with 5-point Likert scale choices included in this survey. The Cronbach’s alpha (coefficient) for the overall questionnaire was 0.82. Calculation of alpha after eliminating one item at a time, showed same or lower than the original alpha value. Table 3 shows the correlation matrix for the 21 questions. The strongest positive correlation was observed between questions Q4 *(I feel confident that the general public are able to assess the legitimacy of claims made by health advertising*) and Q5 (*Testimonials and reviews about health services are helpful for me to make decisions about health services*) (*r* = 0.49, *p* < 0.01). Q4 also demonstrated significant correlation with Q3 (*I feel confident in my abilities to assess the legitimacy of claims made by health advertising*) (*r* = 0.45, *p* < 0.01), Q6 (*I feel the advertising of health services shouldn’t need to be regulated to a greater extent than other types of products or services because health professionals are trustworthy*) (*r* = 0.45, *p* < 0.01) and Q7 (*Health advertising is regulated at an effective level to prevent misleading and deceptive claims*) (*r* = 0.43, *p* < 0.01).

**Table 2:** Percentage responses to survey questions

Question		Percentage Responses (%) *n* = 1564				
		Strongly disagree	Somewhat disagree	Neither agree nor disagree	Somewhat agree	Strongly agree
Q1	Practitioners who use the title “Dr” are only able to do so because they have greater levels of knowledge, skill and professionalism in their practices	3.13	4.73	11.13	36.96	44.05
Q2	There are strict rules in place that govern who is able to use the title “Dr”	1.02	4.92	13.75	37.15	43.16
Q3	I feel confident in my abilities to assess the legitimacy of claims made by health advertising	1.28	6.01	21.55	48.79	22.38
Q4	I feel confident that the general public are able to assess the legitimacy of claims made by health advertising	4.54	18.80	23.91	38.30	14.45
Q5	Testimonials and reviews about health services are helpful for me to make decisions about health services	3.39	7.48	23.79	46.23	19.12
Q6	I feel the advertising of health services shouldn’t need to be regulated to a greater extent than other types of products or services because health professionals are trustworthy	15.92	25.38	21.99	24.55	12.15
Q7	Health advertising is regulated at an effective level to prevent misleading and deceptive claims	3.32	13.24	25.51	40.09	17.84
Q8	Advertising health services based on price (for example – “no gap”) is degrading to the service and presents poorly to the public	8.57	21.23	34.34	25.51	10.36
Q9	Health advertising should distinguish between services that primarily improve health and services which promote cosmetic improvements	0.83	3.32	17.26	42.20	36.38
Q10	Health advertising helps me to understand the costs of my treatment	6.65	17.20	27.30	36.06	12.79
Q11	Health advertising helps to accurately inform the public about available health services	2.43	8.06	22.78	47.15	19.58
Q12	I sometimes feel confused by how relevant advertising for some health services is to my own health needs	3.52	11.32	27.88	42.90	14.39
Q13	The public should not rely on online reviews and testimonials for health services in the same way they might for other products and services (ie hotels and restaurants) because health is more important	1.28	8.63	22.38	36.57	31.14
Q14	Word of mouth recommendations from someone you know is more reliable than online reviews and testimonials	1.79	8.76	25.19	44.18	20.08
Q15	Preferred providers are chosen by healthfunds and insurers because they are the best providers	6.84	21.04	35.49	26.53	10.10
Q16	Preferred providers are chosen by healthfunds and insurers because they have agreed to provide care at a more competitive price	1.85	7.74	30.56	42.71	17.14
Q17	I feel confident in my abilities to spot a review about a health service that was written by a person who was actually a patient of the service	3.58	16.75	34.91	35.17	9.59
Q18	Qualifications should not be displayed by practitioners unless they are directly relevant to the health services that they offer	6.07	14.00	22.06	33.31	24.55
Q19	Special offers from health services are helpful to me as they save me money	2.69	7.16	31.39	41.88	16.88
Q20	Health comparison websites are a helpful and trustworthy source of information	4.54	14.26	32.74	37.02	11.45
Q21	I feel confident that I can tell the difference between advertisements that promote scientifically proven treatments and advertisements which promote treatments that have not been scientifically proven	3.20	13.43	26.92	42.14	14.32

**Table 3:** Correlation matrix for Likert scale questions

	Q1	Q2	Q3	Q4	Q5	Q6	Q7	Q8	Q9	Q10	Q11	Q12	Q13	Q14	Q15	Q16	Q17	Q18	Q19	Q20	Q21
Q1	1.00																				
Q2	0.40^**^	1.00																			
Q3	0.26^**^	0.25^**^	1.00																		
Q4	0.23^**^	0.26^**^	0.45^**^	1.00																	
Q5	0.23^**^	0.23^**^	0.34^**^	0.49^**^	1.00																
Q6	0.10^**^	0.08^**^	0.21^**^	0.45^**^	0.35^**^	1.00															
Q7	0.20^**^	0.23^**^	0.28^**^	0.43^**^	0.42^**^	0.39^**^	1.00														
Q8	0.03	0.03	0.12^**^	0.26^**^	0.20^**^	0.31^**^	0.18^**^	1.00													
Q9	0.16^**^	0.16^**^	0.19^**^	−0.03	0.07^**^	−0.12^**^	0.07^**^	0.00	1.00												
Q10	0.18^**^	0.21^**^	0.25^**^	0.36^**^	0.42^**^	0.32^**^	0.37^**^	0.15^**^	0.05^*^	1.00											
Q11	0.23^**^	0.24^**^	0.26^**^	0.35^**^	0.40^**^	0.26^**^	0.41^**^	0.20^**^	0.11^**^	0.50^**^	1.00										
Q12	0.08^**^	0.03	0.01	0.06^*^	0.08^**^	0.11^**^	0.03	0.21^**^	0.12^**^	0.06^*^	0.05^*^	1.00									
Q13	0.15^**^	0.15^**^	0.11^**^	−0.02	−0.06^*^	−0.01	0.04	0.05	0.26^**^	− 0.01	0.03	0.21^**^	1.00								
Q14	0.08^**^	0.11^**^	0.07^**^	0.09^**^	0.11^**^	0.06^*^	0.11^**^	0.09^**^	0.10^**^	0.10^**^	0.13^**^	0.17^**^	0.19^**^	1.00							
Q15	0.20^**^	0.14^**^	0.19^**^	0.39^**^	0.36^**^	0.39^**^	0.36^**^	0.27^**^	−0.08^**^	0.39^**^	0.35^**^	0.11^**^	0.04	0.16^**^	1.00						
Q16	0.11^**^	0.12^**^	0.17^**^	0.13^**^	0.16^**^	0.07^**^	0.14^**^	0.09^**^	0.16^**^	0.17^**^	0.18^**^	0.13^**^	0.17^**^	0.16^**^	0.20^**^	1.00					
Q17	0.11^**^	0.10^**^	0.31^**^	0.31^**^	0.35^**^	0.30^**^	0.29^**^	0.27^**^	0.04	0.33^**^	0.27^**^	0.11^**^	−0.02	0.09^**^	0.31^**^	0.18^**^	1.00				
Q18	0.07^**^	0.07^*^	0.10^**^	0.08^**^	0.08^**^	0.14^**^	0.08^**^	0.12^**^	0.14^**^	0.10^**^	0.11^**^	0.16^**^	0.20^**^	0.15^**^	0.11^**^	0.14^**^	0.08^**^	1.00			
Q19	0.21^**^	0.20^**^	0.21^**^	0.27^**^	0.43^**^	0.20^**^	0.28^**^	0.07^**^	0.11^**^	0.36^**^	0.40^**^	0.11^**^	0.02	0.13^**^	0.31^**^	0.22^**^	0.28^**^	0.20^**^	1.00		
Q20	0.20^**^	0.15^**^	0.24^**^	0.32^**^	0.43^**^	0.29^**^	0.35^**^	0.15^**^	0.04	0.42^**^	0.37^**^	0.10^**^	−0.05^*^	0.06^*^	0.39^**^	0.19^**^	0.35^**^	0.09^**^	0.43^**^	1.00	
Q21	0.11^**^	0.11^**^	0.38^**^	0.22^**^	0.27^**^	0.17^**^	0.23^**^	0.17^**^	0.12^**^	0.26^**^	0.21^**^	0.04	0.05^*^	0.08^**^	0.20^**^	0.15^**^	0.46^**^	0.10^**^	0.21^**^	0.31^**^	1.00

Spearman correlation coefficients are presented in the table

**. Correlation is significant at the 0.01 level; *. Correlation is significant at the 0.05 level

### Use of titles and qualifications

Eighty-one percent of participants agreed that the use of the title of “Dr” by practitioners was a sign of quality, asserting the practitioners have greater levels of skills, knowledge and professionalism within their practices. This statement was followed by the incorrect assertion that the use of the title “Dr” was strictly regulated which 80.3% of participants agreed with. Over half of participants (57.9%) agreed that only qualifications which were relevant to a practitioner’s professional role should be displayed within advertising (Table 2).

### Confidence in assessing claims made by health advertising

The results found that participants were more likely to be confident in their own abilities (71.2% agreeing in total, with 22.4% agreeing strongly) compared to the ability of the public (52.8% agreeing in total, with 14.5% agreeing strongly). Just over half of participants (56.5%) agreed that they felt confident in their abilities to judge whether claims made in advertising were based on scientific evidence (Table 2). The responses for this question demonstrated a significant positive correlation with the education level of the participants (*r* = 0.11, *p* < 0.01) (Table 4).

**Table 4:** Summary of the bivariate analysis with demographic characteristics

Question		Spearman Correlation Coefficient		
		Age	Education level	Gender
Q1	Practitioners who use the title “Dr” are only able to do so because they have greater levels of knowledge, skill and professionalism in their practices	−0.04	− 0.04	0.02
Q2	There are strict rules in place that govern who is able to use the title “Dr”	0.12^**^	0.00	0.02
Q3	I feel confident in my abilities to assess the legitimacy of claims made by health advertising	−0.04	0.07^**^	0.00
Q4	I feel confident that the general public are able to assess the legitimacy of claims made by health advertising	−0.13^**^	− 0.05^*^	− 0.03
Q5	Testimonials and reviews about health services are helpful for me to make decisions about health services	−0.22^**^	0.04	0.06^*^
Q6	I feel the advertising of health services shouldn’t need to be regulated to a greater extent than other types of products or services because health professionals are trustworthy	−0.17^**^	− 0.03	− 0.06^*^
Q7	Health advertising is regulated at an effective level to prevent misleading and deceptive claims	−0.11^**^	− 0.04	− 0.02
Q8	Advertising health services based on price (for example – “no gap”) is degrading to the service and presents poorly to the public	−0.24^**^	0.04	−0.07^**^
Q9	Health advertising should distinguish between services that primarily improve health and services which promote cosmetic improvements	0.12^**^	0.05^*^	−0.02
Q10	Health advertising helps me to understand the costs of my treatment	−0.15^**^	0.01	0.02
Q11	Health advertising helps to accurately inform the public about available health services	−0.12^**^	− 0.04	0.06^*^
Q12	I sometimes feel confused by how relevant advertising for some health services is to my own health needs	−0.08^**^	0.02	−0.03
Q13	The public should not rely on online reviews and testimonials for health services in the same way they might for other products and services (ie hotels and restaurants) because health is more important	0.17^**^	−0.05	−.065^*^
Q14	Word of mouth recommendations from someone you know is more reliable than online reviews and testimonials	0.05^*^	−0.01	0.01
Q15	Preferred providers are chosen by healthfunds and insurers because they are the best providers	−0.20^**^	− 0.03	− 0.02
Q16	Preferred providers are chosen by healthfunds and insurers because they have agreed to provide care at a more competitive price	−0.06^*^	0.07^**^	−0.01
Q17	I feel confident in my abilities to spot a review about a health service that was written by a person who was actually a patient of the service	−0.28^**^	0.08^**^	−0.03
Q18	Qualifications should not be displayed by practitioners unless they are directly relevant to the health services that they offer	0.00	0.01	−0.09^**^
Q19	Special offers from health services are helpful to me as they save me money	−0.21^**^	0.08^**^	0.04
Q20	Health comparison websites are a helpful and trustworthy source of information	−0.21^**^	0.05^*^	0.01
Q21	I feel confident that I can tell the difference between advertisements that promote scientifically proven treatments and advertisements which promote treatments that have not been scientifically proven	−0.13^**^	0.11^**^	−0.07^**^

Likert scale; 1 = Strongly disagree, 2 = Somewhat disagree, 3 = neither agree nor disagree, 4 = somewhat agree, 5 = strongly agree. A positive correlation with age means when age increases the agreement increases and a positive correlation with education level means the agreement increases with higher education level (Less than Year 12 or equivalent = 1, Year 12 or equivalent (HSC/Leaving certificate) = 2, Vocational Qualification = 3, Associate Diploma = 4, Undergraduate Diploma = 5, Bachelor degree (including honours) = 6, Masters degree = 7, Postgraduate Diploma = 8, Postgraduate Diploma (includes graduate certificate) = 9, Doctorate = 10). For gender, a positive correlation means females agree more than males and vice a versa. ** Correlation is significant at the 0.01 level; * Correlation is significant at the 0.05 level

There was strong agreement that testimonials were not reliable and should not be used in the same way as they are in assessing whether to access other services (67.7%), with 44.8% of participants agreeing that they felt confident in spotting a review that was actually written by a service user. Younger participants (*r* = − 0.28, *p* < 0.01) and participants with higher education reported they felt confident (*r* = 0.08, *p* < 0.01) (Table 4). Over half of the participants agreed that word of mouth recommendations from a known individual were more reliable than testimonials (64.3%), with 48.5% of participants agreeing that healthcare comparison websites were a trustworthy source of information (with 18.8% disagreeing) (Table 4).

### Confusion relating to health advertising

Participants reported that they found themselves confused by how relevant health advertising was to their particular needs (57.3%) and were agreement (78.6%) with the statement: “Health advertising should distinguish between services that primarily improve health and services which promote cosmetic improvements” (Table 2).

### Benefits of health advertising for consumers

The survey participants indicated that health advertising was beneficial to them, with the results showing that participants agreed that advertising helped understand the health services available to them (66.7%), that testimonials were helpful to decision making (65.4%) and that special offers were positive as they helped participants to save money (58.8%). Participants were in agreement that advertising helped them to understand the cost of health services (48.9%) and this was accompanied by 35.9% of participants who agreed that advertising a health service based primarily on price was degrading to the service (compared to 29.8% who disagreed) (Table 2).

### Knowledge about preferred providers

Participants were asked about their understanding about the reasons why practitioners are selected as preferred providers by insurers and health funds; 36.6% agreed that this was because they were the best providers (with 27.9% disagreeing) and 59.9% agreeing that preferred providers were selected due to having agreed to accept a competitive price (with 9.6% disagreeing) (Table 2).

### Regulation of health advertising

Over half of participants felt that health advertising was regulated at an appropriate level (57.9%) but there was a lack of agreement between participants in relation to the statement; “I feel the advertising of health services shouldn’t need to be regulated to a greater extent than other types of products or services because health professionals are trustworthy”, with greater disagreement (41.3%) with this assertion than agreement (36.7%) (Table 2).

## Discussion

Through this research we provide significant insights of how advertising exists within the paradigm of health services and how marketing in healthcare may influence consumer decisions. Participants were asked about the use of the title “Dr” and their perceptions of its use. Under the National Law in Australia, the title of “Dr” is not protected, with practitioners being advised that should they choose to use “Dr”, they should ensure that they list their registered profession, i.e. *Dr John Smith (Chiropractor)*. Traditionally, only medical practitioners and academics with doctorates were recognised as having appropriate justification to use the “Dr”. Over time other professional groups have also adopted “Dr” as an honorific title; for example, in 1983 the Australian Dental Association NSW Branch funded legal action in the NSW Supreme Court successfully prosecuting the issue of whether the Dental Board of NSW could permit dentists to use the title of “Dr” [20]. This finding relating to the title of “Dr” and how the public perceive the connotations and meanings behind the title, suggest that future regulatory policy around health advertising might consider how use of “Dr” may mislead the public.

Participants were asked to indicate how confident they felt they were in their skills in assessing the legitimacy of claims, and how confident they were of the general public’s ability to do the same. The results may be suggestive of a generalised overconfidence of some individual participants in their ability to assess the claims made by health advertising, which could lead to a vulnerability to being misled. The results show that over half of participants (56.5%) agreed that they felt confident in their abilities to judge whether claims made in advertising were based on scientific evidence, with confidence being positively correlated with having attained a higher educational level. The results of our research may illustrate the Dunning-Kruger effect; where the public overestimate their abilities in assessing health claims made by advertising and in doing so then are at risk of making regrettable choices, having drawn incorrect conclusions, therefore being robbed of the ability to recognise this through their incompetence [21]. This overconfidence has explained opposition to conventional medical advice and knowledge, with research showing overconfidence in knowledge relating to health issues being linked to anti-vaccination beliefs [22].

Previous research has shown that consumers use testimonials to help them save time in decision-making [23]. The results from this research are indicative of the participants having consumer attitudes towards health services, they also show that in some instances, health consumers are not motivated by cost and advertising by price is seen by some as being distasteful. Factors surrounding advertising that enhance the amount of information available to the public, such as availability and the experiences of others, had greater perceived value, possibly due to their nature of being empowering, rather advertising based on cost which focuses away from factors such as quality and safety. Participants were aware of the reason for practitioners to be selected as preferred providers by insurers and health funds with participants agreeing that selection was based around practitioners having agreed to accept a competitive fee for their services. However, only 27.9% of participants disagreed with the assertion that being selected to be a preferred provider was a sign of quality. This suggests that the advertisement of preferred provider-status by practitioners and insurers may be misleading to the public. The funders of healthcare services must ensure that they are clear about how providers are chosen to be preferred providers and should consider whether a change in nomenclature could result in less confusion around the role of arrangements that are primarily about securing cheaper rates for health insurers.

While advertising does have the potential to be empowering, the results from this research also suggest that some health consumers are confused by some healthcare adverts and how they relate to their own needs. Berkowitz [24] discusses that the engagement of wants and needs through health advertising is not unethical, providing this occurs in a manner which meets accepted practices. However, if advertising focuses upon creating of patient wants in order to sell more treatment, it is clearly a very different objective to advertising available services. The creation of confusion through the omnipresence of healthcare advertising shows that advertising is not always an empowering influence for consumers.

While there was agreement that healthcare advertising was regulated at an appropriate level, there was a lack of agreement on the subject of health practitioners’ trustworthiness being enough to self-regulate practice in healthcare advertising. This reflected the responses within the qualitative arm of this research where some participants reported being deferential to medical and health professionals, holding a sincere belief in their altruism, with others reporting being distrustful of professional authority. Whilst participants reported finding advertising (including testimonials) helpful, the majority of participants agreed that testimonials should not be relied upon in the same way as they might be for other services (restaurants or hotels), with only a small majority of participants agreeing that they felt able to spot a ‘fake’ review. These results deviate from the conclusions of previous research which suggested that consumers in the tourism industry were becoming savvy in being able to spot ‘fake’ reviews about hotels and restaurants [25]. This finding supports the assertion that the use of testimonials in healthcare should be scrutinised further based on health being based on a different value system to other non-health services. While testimonials can be viewed as an attempt to democratise the environment of healthcare [26], it would be naïve to assume that those who market healthcare services are concerned primarily with patient empowerment rather than selling services. Patients may write and post online reviews for a plethora of reasons; it should not be forgotten that the structures that support this activity are commercially driven. An act of exercising one’s empowerment may in fact equate to contributing to mechanisms that are oriented to exploit the public, detrimentally impacting their autonomy, rather than empowering them [25, 27].

The relative social distance between those who leave online reviews and those who use them, compared to traditional word of mouth from known individuals, makes online testimonials harder to evaluate [28]. Despite this, there was agreement from the majority of participants that online comparison platforms were a trustworthy source of information. In August 2020, following the Australian Competition and Consumer Commission (ACCC) instigating a claim in the Federal Court of Australia against Australia’s largest medical online booking platform, HealthEngine Pty Ltd., for allegedly manipulating patient reviews, the Court ordered the company to pay $2.9 million. The Chair of the ACCC stated, “The ACCC was particularly concerned about HealthEngine’s misleading conduct in connection with reviews it published, because patients may have visited medical practices based on manipulated reviews that did not accurately reflect other patients’ experiences,” [29]. The results of this research demonstrate that the behaviour of platforms that compare health professionals and display patient reviews hold trust with the public and are highly likely to influence patient decision-making. If platforms purporting to provide consumers with a tool to aid their choices do not meet expectations of trustworthiness, they have significant potential to mislead the public.

The sampling of participants was stratified by age and gender. The sample, and therefore the results, cannot claim to be fully representative of the Australian public. However, given this is the first collection of data that examines a cross-section of the Australian public’s views and attitudes on health advertising, the results of this study are informative and helpful in providing an evidence-base to support future policy development in this area.

This research has demonstrated that patients appreciate the perceptions of empowerment that health advertising pretends to give them. Analyses of publicly expressed views on advertising in Australian healthcare reveal that proponents of healthcare marketing value the information and increased participation in healthcare choices that advertising affords [4, 6]. However, health advertising is not provided primarily as a tool for patient empowerment; it is primarily oriented towards the engagement, and in some cases creation, of healthcare consumer needs and therefore may give rise to moral concerns [4, 30]. The participants indicated that they felt health advertising was helpful to them, although the results also suggest that information provided by health advertising may be confusing and not easily assessed by the public for veracity and relevance. Testimonials about health services were reported to be especially difficult to assess. Whilst consumers may find discussing their care online to be helpful and empowering, it is difficult for other members of the public to engage with testimonials about health services in an objective manner. There was strong agreement from the participants that testimonials should not be used for evaluating healthcare in the same way as they might be for evaluating a hotel or a restaurant. The purpose of the consumer discourse within healthcare is to support the individual receiving care to behave as a more significant social and political actor [31]. Testimonials only further this position in a superficial way; online reviews of healthcare services help to further the range of the consumer voice, but this research shows that these messages are difficult to translate into real meaning for other consumers. If health consumers are to be truly empowered by digital technology, this should occur through comprehensive access to information about clinical services, collected and presented systematically, allowing consumers to base their clinical choices on real data about the quality and efficacy of services. Having the ability to choose a restaurant based on whether another consumer enjoyed their experience might be helpful, however the participants in this research indicated that health is more important, making this approach fraught when applied to healthcare services. The way in which practitioners present themselves has also been shown by this study to be important, with, for example, this research revealing a large disparity between public perceptions of the title “Dr” and the reality of its use and regulation.

## Conclusions

The knowledge created by this research will be available to influence the future direction of policy in this area; the Independent Review of the National Registration and Accreditation Scheme for health professions recommended: “Make amendments to the Health Practitioner Regulation National Law 2009 provision preventing the use of testimonials on platforms and sites that are managed or controlled by the practitioner or business.” [6]. This was the only recommendation in relation to health advertising that was made. This research discusses how other elements of health advertising may contribute to consumer choices about which health services to access, as well as how health consumers perceive and engage with advertising in the health context. We, the authors, hope that healthcare and commercial regulators, along with healthcare professional associations consider these findings and their ramifications for the future conduct of healthcare practise.

Health advertising may be helpful to health consumers, and its presence is to be accepted in the commercialised world of the twenty-first Century. However, the framework surrounding how the public are protected from health professionals, both registered and unregistered, may need to be reconsidered and modified in order to maintain public safety in a world where advertising is as rapidly changing as the technology the enables it and the society which consumes it. These results demonstrate that health consumers may find advertising helpful and may be superficially empowering. However, this research also illustrates that the public are vulnerable to misinformation and predatory commercial behaviours, with a significant number of consumers being unable to evaluate many aspects of health advertising.

## Supplementary Information

**Additional file 1.** Survey Questions.

## Data Availability

The datasets used and analysed during the current study available from the corresponding author on reasonable request.

## Abbreviations

NSW: New South Wales
AHPRA: Australian Health Practitioner Regulation Agency
DAA: Dietitians Association of Australia
ACCC: Australian Competition and Consumer Commission
TGA: Therapeutic Goods Administration
