# Supplementary material for: What do Australian health consumers believe about commercial advertisements and testimonials? a survey on health service advertising
Source: BMC Public Health. 2021 Jan 7;21:74. doi: 10.1186/s12889-020-10078-9 (PMC7791787; doi:10.1186/s12889-020-10078-9)
Supplement: Supplementary file 1 — Additional file 1. Survey Questions. [file 12889_2020_10078_MOESM1_ESM.docx]

**Survey Instrument**

The survey questions were be presented as a Likert scale, with participants selecting between 1 (strongly disagree) and 5 (strongly agree).

1. Practitioners who use the title “Dr” are only able to do so because they have greater levels of knowledge, skill and professionalism in their practices.
2. There are strict rules in place that govern who is able to use the title “Dr”.
3. I feel confident in my abilities to assess the legitimacy of claims made by health advertising.
4. I feel confident that the general public are able to assess the legitimacy of claims made by health advertising.
5. Testimonials and reviews about health services are helpful for me to make decisions about health services.
6. I feel the advertising of health services shouldn’t need to be regulated to a greater extent than other types of products or services because health professionals are trustworthy.
7. Health advertising is regulated at an effective level to prevent misleading and deceptive claims.
8. Advertising health services based on price (for example – “no gap”) is degrading to the service and presents poorly to the public.
9. Health advertising should distinguish between services that primarily improve health and services which promote cosmetic improvements.
10. Health advertising helps me to understand the costs of my treatment.
11. Health advertising helps to accurately inform the public about available health services.
12. I sometimes feel confused by how relevant advertising for some health services is to my own health needs.
13. The public should not rely on online reviews and testimonials for health services in the same way they might for other products and services (ie hotels and restaurants) because health is more important.
14. Word of mouth recommendations from someone you know is more reliable than online reviews and testimonials.
15. Preferred providers are chosen by healthfunds and insurers because they are the best providers.
16. Preferred providers are chosen by healthfunds and insurers because they have agreed to provide care at a more competitive price.
17. I feel confident in my abilities to spot a review about a health service that was written by a person who was actually a patient of the service.
18. Qualifications should not be displayed by practitioners unless they are directly relevant to the health services that they offer.
19. Special offers from health services are helpful to me as they save me money.
20. Health comparison websites are a helpful and trustworthy source of information.
21. I feel confident that I can tell the difference between advertisements that promote scientifically proven treatments and advertisements which promote treatments that have not been scientifically proven.
